# Association between High-density-lipoprotein-cholesterol Levels and the Prevalence of Asymptomatic Intracranial Arterial Stenosis

**DOI:** 10.1038/s41598-017-00596-9

**Published:** 2017-04-03

**Authors:** Xin Li, Anxin Wang, Jing Wang, Jianwei Wu, Dandan Wang, Xiang Gao, Shouling Wu, Xingquan Zhao

**Affiliations:** 1 Department of Neurology, Beijing Tiantan Hospital, Capital Medical University, Beijing, China; 2 China National Clinical Research Center for Neurological Diseases, Beijing, China; 3 Center of Stroke, Beijing Institute for Brain Disorders, Beijing, China; 4 Beijing Key Laboratory of Translational Medicine for Cerebrovascular Disease, Beijing, China; 5 Department of Cardiology, Kailuan Hospital, Tangshan, China; 6 Department of interventional neurology, the affiliated hospital of Qingdao university, Qingdao, China; 7 Department of Nutrition, Harvard University School of Public Health, Boston, MA USA; 8 Channing Laboratory, Department of Medicine, Brigham and Women’s Hospital, and Harvard Medical School, Boston, MA USA

## Abstract

Intracranial atherosclerotic stenosis (ICAS) is a common cause of ischemic stroke, and a low level of high-density lipoprotein cholesterol (HDL-C) is also considered to be a predictor for stroke. However, the association between the HDL-C level and asymptomatic ICAS is uncertain. From 2010 to 2011, a random sample of 5,351 participants were enrolled in the Asymptomatic Polyvascular Abnormalities Community (APAC) study. The recruited participants were then separated into 5 roughly uniform-sized factions with varying HDL-C levels. Multivariate logistic regression was implemented to assess the connection of the HDL-C levels and the prevalence of asymptomatic ICAS. The prevalence of asymptomatic ICAS showed no gradual decrease with the increase of HDL-C levels. After adjustment for conventional risk factors, HDL-C levels still showed no significant association with asymptomatic ICAS. The odds ratios (OR) of the prevalence of asymptomatic ICAS between the first group and the other 4 groups were 0.98, 1.00, 0.92, and 0.87 with 95% confidence intervals (CI) being 0.76–1.27, 0.78–1.29, 0.71–1.19, and 0.66–1.13, respectively. The study showed little correlation between HDL-C levels and asymptomatic ICAS. Normal levels of HDL-C are not an independent risk factor for asymptomatic ICAS.

## Introduction

Intracranial arterial stenosis (ICAS) is one of the most common causes of ischemic stroke^1^, which, in turn, is one of the main causes of morbidity and mortality worldwide, especially in China^2^. The presence of ICAS is associated with a poor prognosis for patients that have experienced ischemic stroke^3^. Chimowitz MI *et al*. found the one-year recurrence rate of ischemic stroke to be 23% in patients with ICAS of more than 70%^4^. Intracranial atherosclerosis is more common in Asian countries than in the United States. In China, approximately 30%–40% of cerebral infarctions (CIs) and more than 50% of transient ischemic attacks (TIAs) are connected to the appearance of ICAS, which is much greater than in Western nations^3^. The racial difference in the distribution of intracranial and extracranial atherosclerosis stenosis is large. Genetic susceptibility might play a critical role in the different pathophysiology observed in different populations. Previous studies have used traditional vascular disease risk factors, including hypertension, diabetes mellitus (DM), smoking, hyperlipidemia, and low-density lipoprotein cholesterol (LDL-C) levels, to determine the prevalence of asymptomatic ICAS^5, 6^.

Previous trials have consistently shown an inverse relationship between the levels of HDL-C and cardiovascular disease (CVD) and stroke^7^. However, even though HDL has properties consistent with atheroprotection, the causal relationship between HDL and atherosclerosis is not clear. After a series of failed human genetics and clinical trials, an increasing number of authors have expressed skepticism about the HDL hypothesis, which holds that raising HDL-C would reduce the risk of atherosclerotic vascular disease^8–11^. Some epidemiological studies have shown an inverse relationship between HDL-C and the risk of stroke^7, 12, 13^, but others have not^14^. This study was conducted to determine whether HDL-C levels are associated with asymptomatic ICAS among Chinese adults.

## Results

### Prevalence of ICAS

In all, 698 cases of ICAS were found among the 5,351 participants, a prevalence of 13.0% (698/5,351). In this study, there were 460 subjects (310 men and 150 women) who did not have a good temporal window for transcranial Doppler (TCD). Of the 5,351 participants, 230 (4.3%) were found to have stenosis in the anterior cerebral artery, 342 (6.4%) in the middle cerebral artery, 58 (1.1%) in the posterior cerebral artery, 66 (1.2%) in the vertebral artery, and 89 (1.7%) in the basal artery. The stenosis affected a variety of arteries, with 355 participants (6.5%) found to have stenosis in 2 or more arteries.

### Baseline Characteristics

The baseline traits of the patients in every quintile HDL-C faction are revealed in Table 1. Median values are presented for all subgroups. The median HDL-C levels were 1.13 mmol/l, 1.35 mmol/l, 1.57 mmol/l, 1.83 mmol/l, and 2.19 mmol/l for quintiles 1 to 5, respectively. Results showed the differences in gender, age, smoking, hypertension, DM, BMI, TC, LDL-C, and triglyceride levels among quintiles to be significant (P < 0.01). The absolute values of the medians of age, BMI, and TG level decreased as quintile value increased, but the absolute value of TC continuously increased as HDL-C levels increased. The male smokers who had hypertension or higher level of LDL-C were more prevalent in the groups with lower HDL-C levels. There were no significant associations between the level of physical activity, DM, FBG, family history of MI or stroke, and HDL-C levels (P > 0.01).

**Table 1:** Median baseline attributes of patients by HDL-C quintiles.

	Group_a					*P v*alue
	Q1	Q2	Q3	Q4	Q5	
HDL-C, median(IQR), mmol/l	1.13 (1.02–1.20)	1.35 (1.30–1.41)	1.57 (1.52–1.63)	1.83 (1.76–1.90)	2.19 (2.07–2.40)	<0.01
Number	1,063	1,058	1,108	1,064	1,058	<0.01
Women, n (%)	347 (32.6)	371 (35.1)	473 (42.7)	477 (44.8)	468 (44.2)	<0.01
Age, median(IQR), years	54.99 (47.77–63.48)	52.32 (45.15–61.14)	52.18 (45.63–61.22)	51.33 (44.96–59.76)	51.27 (45.35–61.01)	<0.01
Smoking, n (%)	379 (35.7)	365 (34.5)	333 (30.0)	325 (30.6)	316 (29.9)	<0.01
Hypertension, n (%)	591 (55.6)	530 (50.1)	520 (46.9)	456 (42.9)	456 (43.1)	<0.01
Diabetes, n (%)	146 (13.7)	122 (11.5)	143 (12.9)	121 (11.4)	104 (9.8)	>0.05
Physical activity,						0.09
Inactive, n (%)	435 (40.9)	433 (40.9)	427 (38.5)	429 (40.3)	425 (40.2)	
Moderately active, n (%)	248 (23.3)	246 (23.3)	318 (28.7)	285 (26.8)	257 (24.3)	
Very active, n (%)	380 (35.8)	379 (35.8)	363 (32.8)	350 (32.9)	376 (35.5)	
Family history of MI, n%	16 (1.97)	8 (0.97)	13 (1.50)	18 (2.14)	19 (2.26)	0.21
Family history of stroke, n (%)	32 (3.93)	21 (2.55)	28 (3.24)	26 (3.09)	21 (2.51)	0.45
BMI, Median (IQR) kg/m^2^	25.71 (23.66–27.77)	25.21 (23.23–27.43)	24.77 (22.70–27.04)	24.24 (22.31–26.44)	23.67 (21.71–25.95)	<0.01
FBG, Median (IQR), mmol/l	5.21 (4.80–5.86)	5.20 (4.83–5.75)	5.23 (4.85–5.81)	5.19 (4.82–5.74)	5.22 (4.84–5.80)	0.65
TC, median (IQR), mmol/l	4.70 (4.13–5.28)	4.80 (4.27–5.47)	4.84 (4.33–5.59)	5.01 (4.48–5.63)	5.43 (4.79–6.15)	<0.01
LDL-C, median (IQR), mmol/l	2.50 (2.01–2.96)	2.54 (2.15–3.00)	2.67 (2.23–3.08)	2.69 (2.27–3.11)	2.59 (2.14–3.12)	<0.01
TG, median (IQR), mmol/l	1.59 (1.13–2.50)	1.38 (1.01–2.06)	1.28 (0.93–1.82)	1.20 (0.85–1.64)	1.07 (0.79–1.66)	<0.01

IQR: interquartile range.

### Connection between Baseline HDL-C Levels and the Prevalence of Asymptomatic ICAS

Without any adjustment for confounders or effect-modifiers, as shown in Table 2, HDL-C does not independently indicate the presence of asymptomatic ICAS. In contrast to the first quintile (Q1), crude OR (95% CI) of the second (Q2), third (Q3), fourth (Q4), and fifth (Q5) quintiles were 0.96 (0.75–1.23), 0.99 (0.77–1.26), 0.89 (0.69–1.15), and 0.84 (0.65–1.08), respectively. After adjusting for gender and age, the estimated correlation between HDL-C and the prevalence of asymptomatic ICAS still yielded negative OR and Model 1 adjusted OR (95% CI) of Q2, Q3, Q4, and Q5 were 0.96 (0.75–1.23), 0.98 (0.77–1.25), 0.88 (0.68–1.14), and 0.83 (0.64–1.07), respectively. Even after adjusting for any possible confounders, the significant correlation between HDL-C levels and asymptomatic ICAS diagnosis was also negative. Model 2 adjusted OR (95% CI) of Q2, Q3, Q4, and Q5 were: 0.98 (0.76–1.27), 1.00 (0.78–1.29), 0.92 (0.71–1.19), and 0.87 (0.66–1.13), respectively. Finally, the difference between stratified selected risk factors and the interaction effects on the association were analyzed further to estimate the exact correlation between the prevalence of asymptomatic ICAS and the HDL-C level. Results indicated there to be no positive interaction between the effect of HDL-C levels and the subgroup factors. The outcome of the trend test for diabetes was 0.04, indicating there could be a dose-reliant connection linking serum HDL-C quintiles and the prevalence of ICAS in diabetes. However, the entire 95% confidence interval of the odd ratios for ICAS in diabetes based on HDL-C level quintiles contains 1.0. The multivariate-adjusted OR (95% CI) of Q2, Q3, Q4, and Q5 in diabetes were: 0.92 (0.52–1.64), 0.80 (0.46–1.38), 0.72 (0.40–1.30), and 0.53 (0.28–1.01), respectively (Table 3).

**Table 2:** Ratio of odds (95% confidence interval) for ICAS based on baseline HDL-C level quintiles.

	Median	ICAS n (%)	Crude OR (95% Cl)	Model 1 (95% Cl)	Model 2 (95% Cl)
HDL-C					
Q1	1.13	154 (14.49)	1	1	1
Q2	1.35	138 (13.04)	0.96 (0.75–1.23)	0.96 (0.75–1.23)	0.98 (0.76–1.27)
Q3	1.57	152 (13.72)	0.99 (0.77–1.26)	0.98 (0.77–1.25)	1.00 (0.78–1.29)
Q4	1.83	128 (12.03)	0.89 (0.69–1.15)	0.88 (0.68–1.14)	0.92 (0.71–1.19)
Q5	2.19	126 (11.91)	0.84 (0.65–1.08)	0.83 (0.64–1.07)	0.87 (0.66–1.13)
*P* for trend			0.15	0.12	0.24

HDL-C: high-density lipoprotein cholesterol.

*Model 1: adjusted for gender and age.

Model 2: adjusted for gender, age, hypertension, DM, BMI, SBP, DBP, FBG, smoking, physical activity, family history of myocardial infarction, TC, and TG.

**Table 3:** Multivariate-adjusted odd ratios for ICAS based on HDL-C levels, classified by gender and chosen risk elements.

	Q1	Q2	Q3	Q4	Q5	P trend	P interaction
	Group_a						
Men	1	0.99 (0.73–1.36)	0.92 (0.67–1.27)	1.01 (0.73–1.41)	0.92 (0.66–1.29)	0.71	0.66
Women	1	0.95 (0.61–1.47)	1.04 (0.69–1.57)	0.73 (0.47–1.14)	0.75 (0.48–1.16)	0.091	
Age < 60	1	1.15 (0.81–1.63)	1.16 (0.82–1.63)	1.07 (0.75–1.52)	0.88 (0.61–1.28)	0.44	0.41
Age ≥ 60	1	0.83 (0.56–1.22)	0.84 (0.58–1.23)	0.74 (0.49–1.10)	0.86 (0.58–1.28)	0.32	
Non-hypertension	1	0.78 (0.49–1.23)	1.00 (0.65–1.53)	0.75 (0.48–1.18)	0.92 (0.59–1.44)	0. 70	0.41
Hypertension	1	1.09 (0.80–1.48)	0.98 (0.72–1.33)	1.00 (0.73–1.38)	0.80 (0.57–1.12)	0.20	
Non-diabetes	1	1.00 (0.75–1.33)	1.05 (0.80–1.40)	0.97 (0.72–1.30)	0.95 (0.71–1.28)	0.72	0.59
Diabetes	1	0.92 (0.52–1.64)	0.80 (0.46–1.38)	0.72 (0.40–1.30)	0.53 (0.28–1.01)	0.04	
BMI < 30 kg/m^2^	1	1.00 (0.76–1.30)	1.03 (0.79–1.34)	0.92 (0.71–1.21)	0.89 (0.68–1.17)	0.34	0.94
BMI ≥ 30 kg/m^2^	1	0.87 (0.35–2.17)	0.66 (0.25–1.74)	0.86 (0.30–2.50)	0.61 (0.16–2.36)	0.43	
Physical activity							0.45
Inactive	1	1.05 (0.70–1.57)	0.91 (0.60–1.39)	1.05 (0.70–1.59)	0.99 (0.65–1.51)	0.97	
Moderately active	1	0.97 (0.55–1.71)	1.11 (0.67–1.87)	1.05 (0.60–1.82)	0.90 (0.51–1.61)	0.86	
Very active	1	0.93 (0.62–1.38)	1.00 (0.67–1.48)	0.73 (0.48–1.13)	0.74 (0.48–1.13)	0.09	

Model 1: Multivariate-adjusted odd ratios (OR): adjusted for gender, age, hypertension, DM, SBP, DBP, FBG, LDL-C, TC, TG, BMI, family history of myocardial infarction, and physical activity.

## Discussion

Stroke has become the leading cause of long-term disability and death in China, with more than 2 million newly diagnosed cases each year^15^. Many studies have shown that ICAS is closely associated with ischemic stroke, especially in Asia^16^. In our previous reports, many known risk factors, including TC, NON-HDL, FBG, and metabolic syndrome, have been shown to be related to the development of ICAS within the APAC population-based cohort^17–20^. However, the correlation between the serum HDL-C levels and ICAS remains to be examined^21^, especially with respect to asymptomatic ICAS. This is the first evaluation to offer proof demonstrating that there is little to no link between serum HDL-C and the prevalence of asymptomatic ICAS. In the current study, results showed the prevalence of ICAS to be 13.4%, which was significantly higher than previous studies. The most important finding was that the correlation between HDL-C and asymptomatic ICAS remains uncertain in both male and female participants. No significant difference was established in the prevalence of asymptomatic ICAS between quintiles, even after adjusting for potential confounding factors. These results suggest that the levels of serum HDL-C might not be as important to the identification of asymptomatic ICAS as has been thought.

The present study was a community-based large epidemiological survey. In this way, magnetic resonance angiography (MRA) and digital subtraction angiography (DSA) were found to be unsuitable. As for the identification of ICAS, the sensitivity and specificity of TCD were 91.4% and 82.7%, respectively^22^. Because TCD is simple, non-invasive, convenient, and highly repeatable, it is currently regarded as the optimal indicator of the consecutive monitoring of intracranial hemodynamics^23^.

HDL-C is considered a principal element in metabolic syndrome by the International Diabetes Federation and National Cholesterol Education Program. The inverse relationship between the risk of cardiovascular disease, including ischemic stroke, and the serum level of HDL-C concentration was documented using epidemiological data and clinical trials^12, 24–26^. Yining Qian *et al*. scrutinized the connection between the concentration of HDL-C and risk of having ICAS in acute ischemic stroke patients and found a negative connection between HDL-C levels and the risk of ICAS^27^. Glomset *et al*. proposed the HDL hypothesis, which states that HDL can promote the reverse transport of cholesterol and that increasing HDL-C can reduce the risk of coronary heart disease^28^, a hypothesis that has been supported by a series of preclinical studies. However, not all of the studies have been consistent with the HDL hypothesis. Randomized controlled trials and human genetics studies have shown that HDL-C is not always predictive of cardiovascular disease^8–11^.

Results showed the prevalence of ICAS to be 13.4%, which is higher than has been reported in other trials. Meseguer *et al*. demonstrated that the prevalence of symptomatic ICAS was 8.8% in France^29^. Tsivgoulis *et al*. examined Caucasian patients who had acute CI and discovered the rate of symptomatic ICAS to be 9.2%^30^. A prior evaluation performed in China in 2007, with an identical Chinese population and the exact same diagnostic criteria for ICAS revealed a rate of asymptomatic ICAS of 6.9%^31^. The alterations in the prevalence of ICAS over the last 4 years could be linked to the rise in the occurrence of dyslipidemia and related cardiovascular diseases in current-day China^32, 33^.

In the current study, all participants were classified into 5 groups according to the serum HDL-C quintiles. Results showed that the difference in the prevalence of ICAS across the HDL-C quintiles was not significant. In the current study population, there were no inverse quantitative interactions between the prevalence of asymptomatic ICAS and the HDL-C concentration. More and more studies have come to focus on the association between plasma lipid levels and the presence of ICAS, indicating that hyperlipidemia, LDL-C, hypercholesterolemia, TC, and NON-HDL are all independent risk factors for ICAS^3, 6, 18–20^. Yining Qian *et al*. revealed low HDL-C levels to be linked with the growth of ICAS in Chinese patients who had experienced acute ischemic stroke and proposed that the level of HDL-C and the risk of having ICAS was inversely associated, which was notably different from the contemporary outcomes^27^.

The differences in these results are not surprising considering that the study by Yining Qian *et al*. examined ICAS in acute ischemic stroke patients, while the participants in the current study were asymptomatic and selected from the general population. One possible hypothesis that might reconcile the results of these reports is that HDL-C levels may be predictive of symptomatic ICAS but not asymptomatic ICAS. Bum Joon Kim *et al*. found that the progression of symptomatic ICAS differs from that of asymptomatic ICAS and that their predictors differ accordingly. They detected the level of HDL-C and found it to be associated with changes in the status of symptomatic ICAS but independent of changes in asymptomatic ICAS^34^. Some ICAS of non-atherosclerotic origin may have been included in the current study, and this could be another factor that might explain the negative results.

In the current study, the median of the first quintile was 1.1 mmol/l. The HDL-C concentration of most participants was higher than the HDL-C standard level, 1.04 mmol/l. In this context, the relatively normal level of HDL-C might be another reason to explain why the prevalence of asymptomatic ICAS and the HDL-C are independent from each other. In light of the current study, it can be inferred that a normal level of HDL-C was not a predictor of asymptomatic ICAS.

The HDL hypothesis was challenged by data derived from randomized controlled trials of HDL-increasing drugs and human genetics studies. Considerable effort has been devoted to determining the mechanism underlying the physiological regulation and functions of HDL-C. Jean-Pierre Després *et al*. reviewed these studies and described the properties of HDL that may prevent the development of atherosclerosis^35^: (a) cholesterol efflux from macrophages in the artery wall, (b) anti-inflammatory activity, (c) antidiabetic activity, (d) antithrombotic activity, (e) endothelial repair and improved function, (f) promotion of angiogenesis, (g) vasodilatory activity, and (h) antioxidative activity. With the development of clinical laboratory technology, the various properties of HDL, including size, composition, metabolomics, migration on 2D gels, the role of cholesterol efflux, and the ability to act as antioxidants, have been examined. In the current study, HDL-C is treated as one feature of HDL, and is considered to be reflective solely of the cholesterol of partial HDL fraction that is most often separated by precipitation techniques. It is not an adequate indictor of HDL function. That might be the fourth reason why no inverse relationship was here observed between the level of HDL-C and the prevalence of asymptomatic ICAS.

The current study has several limitations. First, although the APAC study is a prospective, long-term follow-up observational study, no follow-up data were available. For this reason, the current analysis was designed as a cross-sectional evaluation. Long-term outcomes will be reported in subsequent studies.

Second, participants with inadequate temporal windows for TCD analysis were considered non-ICAS, which may have caused the prevalence of asymptomatic ICAS to be underestimated. Third, the original Kailuan study had an imbalanced gender distribution, and male participants far outnumbered female ones. In order to overcome this limitation, the female ratio in the APAC study was increased during the random selection process. Notwithstanding these restrictions, this evaluation is significant because there have not been any other works that have analyzed the association between HDL-C levels and the prevalence of asymptomatic cerebral stenosis in a large-population cohort. Further studies should be performed to confirm these findings.

## Methods

### Population and Study Design

**Figure 1:**
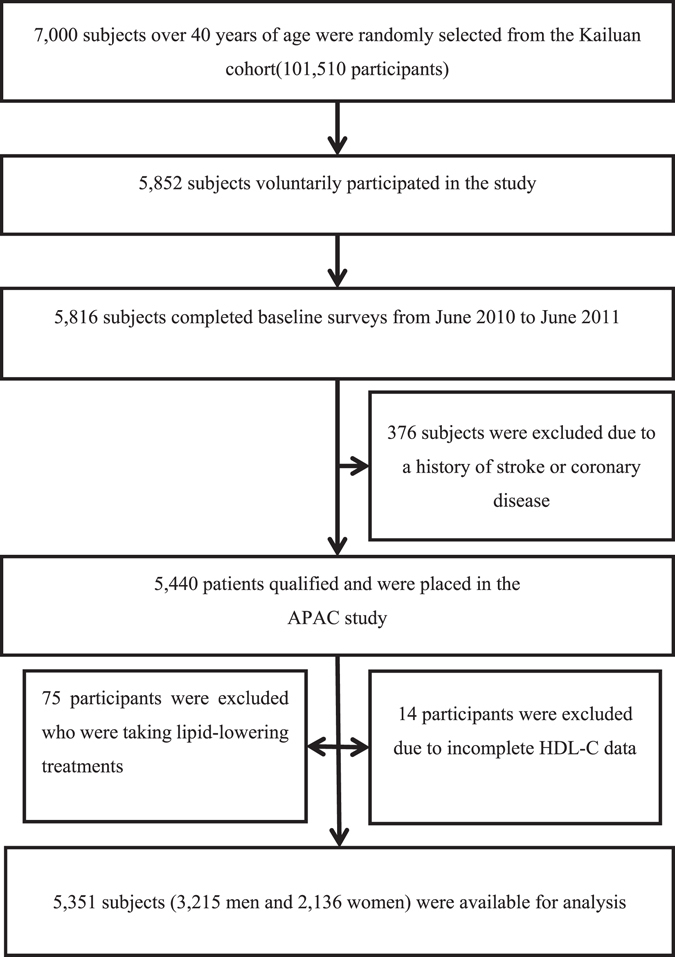
Flow chart of the method.

The APAC is a prospective, community-based, long-term follow-up observational study^32^. It was performed to evaluate the epidemiology of carotid atherosclerosis, asymptomatic ICAS, and diseases of the peripheral artery in Chinese adults. A representative portion of 7,000 participants at least 40 years old were arbitrarily selected from a control community of 101,510 subjects (81,110 men and 20,400 women, 18 to 98 years of age) in a Kailuan legion^32^ with stratified arbitrary sampling via age and gender according to information from the 2010 census. The Kailuan evaluation was a continuous expected evaluation started in 2006 performed on a Kailuan populace in Tangshan City, a large, modern coastal city southeast of Beijing. This community was chosen because of their outstanding responsiveness and good reputation for making follow-up appointments. The Kailuan population also involves people with diverse occupations from a variety of social rankings. The representative population was calculated according to the determination of 7% of the event rate with 0.7% precision, alpha = 0.05. The response rate was believed to be 80%. The first cohort of 5,852 participants was enrolled, and 5,816 completed the baseline examination and evaluation between June 2010 and June 2011. Of these 5,816 potential participants, 376 did not meet the inclusion criteria, which were as follows: no history of stroke, transient ischemic attack, or coronary disease at baseline as evaluated by a substantiated survey and a lack of neurologic shortfalls suggesting prior stroke as discovered by investigation by qualified doctors. Throughout the baseline review, the entire group completed questionnaire assessment and underwent clinical, laboratory, and TCD evaluations. Here 14 subjects with deficient HDL-C data and 75 subjects who were taking lipid-lowering regimens were eliminated. This left 5,351 subjects (3,215 men and 2,136 women) in the analyses available for final analysis in this study (Fig. 1). There was no substantial difference in the fundamental traits of the registered participants and those who were eliminated (P > 0.05). This protocol was approved by the Ethics Committees of Beijing Tiantan Hospital and Kailuan General Hospital and performed in accordance with the guidelines of the Helsinki Declaration. Written informed consent was gained from all subjects and the study was authorized by the relevant Ethics Committees.

### Measurement of Indicators

A questionnaire was used to collect the baseline information on the enrolled subjects, including gender, age, family history of cardiovascular disease, menopausal status, smoking status, alcohol habits, hypertension, hyperlipidemia, DM, and medications prescribed by physicians. During the baseline interview, anthropometric indices included height, weight, and blood pressure (BP). The BP listed here is the average of two readings taken at rest. If the two differed by more than 5 mmHg, another reading was taken and the average of the 3 was used. Body mass index (BMI) was calculated as body weight (kg) divided by the square of body height (m^2^). The participants were further characterized based on specific parameters, i.e., gender, age, BMI, and physical activity.

Blood samples were drawn from the antecubital vein during the baseline interview after an overnight fast and collected in vacuum tubes containing ethylenediaminetetraacetic acid (EDTA). The tubes were then centrifuged for 10 min at 3,000 rpm at room temperature. Then the plasma samples were prepared and analyzed within 4 h of preparation. Cholesterol and triglyceride were measured enzymatically (Mind Bioengineering Co. Ltd., Shanghai, China). The hexokinase/glucose-6-phosphate dehydrogenase method was used to measure the fasting blood glucose (FBG). All blood samples were analyzed using an autoanalyzer (Hitachi 747; Hitachi, Tokyo, Japan) at the central laboratory of Kailuan General Hospital.

### Definitions

Hypertension was defined as the existence of any of the following: a prior history of identified hypertension, current regimen of antihypertensive therapy, systolic blood pressure ≥ 140 mmHg, or a diastolic pressure ≥ 90 mmHg. DM was defined as the presence of any of the following: a prior history of DM or current oral hypoglycemic treatment or insulin regimen, or FBG levels greater than 7.0 mmol/l. Hypercholesterolemia was defined as the presence of any of the following: a prior history of hypercholesterolemia, current regimen of cholesterol-lowering therapy, or total cholesterol level of greater than 5.17 mmol/l.

### Assessment of ICAS

TCD was chosen to quantify arterial stenosis due to its noninvasiveness, efficiency, and dependability in the identification of intracranial arterial stenosis^36^, and the simplicity of application to big assemblies of people. TCD was performed by 2 trained radiologists with portable devices (EME Companion, Nicolet) with 2 MHz probes. ICAS identification was made based on the peak flow velocity of standard benchmarks and proven against MR angiography and clinical results^29, 31^. Briefly, ICAS was stipulated as a peak systolic flow velocity (Vp) > 100 cm/s for the posterior cerebral artery and vertebra-basilar artery; >120 cm/s for the anterior cerebral artery and internal carotid siphon; and >140 cm/s for the middle cerebral artery. Additional elements were also taken into account in the identification of ICAS, including patients’ age, the presence of a disturbance in turbulence, echo prevalence, and if the abnormal velocity was subdivided. Undiscovered arteries by way of temporal and orbital windows were regarded as ICAS negative. Participants were categorized with occlusive disease if a minimum of 1 of the examined arteries revealed proof of stenosis or occlusion.

### Statistical Analysis

Statistical analyses were performed using SAS software, version 9.1 (SAS Institute, Cary, NC, U.S.). Two-tailed tests were used in all statistical analyses, and P-values below 0.05 were considered statistically significant. The participants were divided into 5 groups based upon serum HDL-C quintiles. Because the dispersion of all of the constant variables was distorted, the medians were taken for evaluation and compared with analyses of variance (ANOVA) and categorical variables were compared using chi-square tests. Logistic regression models were used to compute the gender- and age-adjusted and multivariate-adjusted odds ratios (ORs) and 95% CI. The multivariate-adjusted model was further adapted for gender, age, hypertension, DM, family history of myocardial infarction and stroke, physical activity, current smoking status, SBP, DBP, BMI, FBG, TC, LDL-C, and triglycerides. A trend test was used to determine whether a dose-reliant connection was apparent between serum HDL-C quintiles and the typical incidents of ICAS. Categorical data were considered constant data with the median value of HDL-C levels in every quintile. Gender and other possible indicators were also examined to determine if there was any specific interplay among these variables and to evaluate the connection between HDL-C levels and the existence of ICAS.

## Conclusion

Asymptomatic ICAS has become more prevalent in China than ever before. Subgroup analysis showed little association between the HDL-C level and asymptomatic ICAS. Typical HDL-C levels were not considered an independent risk component of asymptomatic ICAS.
